# Comparative activity of ceftibuten combinations with avibactam, ledaborbactam, and xeruborbactam against recombinant β-lactamase-producing *Escherichia coli* and contemporary WGS-characterized strains of carbapenemase-producing Enterobacterales

**DOI:** 10.1128/aac.00195-26

**Published:** 2026-05-29

**Authors:** Lucía Sánchez-Peña, Salud Rodríguez-Pallares, Tania Blanco-Martín, Gloria Pérez-Rodríguez, Pablo Aja-Macaya, Lucía González-Pinto, Inmaculada López-Hernández, Belén Aracil García, Alejandro Beceiro, Germán Bou, Jorge Arca-Suárez

**Affiliations:** 1 Servicio de Microbiología Clínica and Grupo de Investigación en Microbiología, Instituto de Investigación Biomédica de A Coruña (INIBIC) and Complexo Hospitalario Universitario de A Coruña (CHUAC), SERGAS. Universidade da Coruña (UDC) 16811, A Coruña, Spain; 2 CIBER de Enfermedades Infecciosas (CIBERINFEC), Instituto de Salud Carlos III 38176, Madrid, Spain; 3 Instituto de Biomedicina de Sevilla IBIS, Hospital Universitario Virgen Macarena/CSIC/Universidad de Sevilla 16778, Seville, Spain; 4 Departamento de Microbiología, Facultad de Medicina, Universidad de Sevilla 73063 , Seville, Spain; 5 Unidad de Microbiología y Enfermedades Infecciosas, Hospital Universitario Virgen, Macarena 16582, Seville, Spain; 6 Laboratorio de Referencia e Investigación en Resistencia a Antibióticos e Infecciones Relacionadas con la Asistencia Sanitaria, Centro Nacional de Microbiología, Instituto de Salud Carlos III 38176, Madrid, Spain; 7 Departamento de Fisioterapia, Medicina y Ciencias Biomédicas, Universidad de A Coruña 16737 , A Coruña, Spain; University of Fribourg, Fribourg, Switzerland

**Keywords:** carbapenemase, β-lactam, β-lactamase inhibitor, β-lactamase, carbapenemase-producing Enterobacterales, Enterobacterales, xeruborbactam, ledaborbactam, avibactam, ceftibuten

## Abstract

We evaluated the activity of ceftibuten combinations with avibactam, ledaborbactam, and xeruborbactam against isogenic *Escherichia coli* and clinical Enterobacterales isolates producing broad-spectrum β-lactamases. Avibactam and ledaborbactam enhanced ceftibuten activity against transformants producing serine-β-lactamases. Ceftibuten/xeruborbactam was also active against VIM and some IMP producers. All combinations were active against OXA-48- and KPC-producing clinical isolates. Ceftibuten/xeruborbactam also retained activity against VIM-producing clinical strains. Susceptibility rates for MBL producers varied, depending on the CLSI or EUCAST breakpoints applied.

## INTRODUCTION

Infections caused by multidrug-resistant (MDR) Gram-negative bacteria, particularly third-generation cephalosporin-resistant and carbapenem-resistant Enterobacterales, are associated with limited therapeutic options and high morbidity and mortality (1). Resistance to third-generation cephalosporins in Enterobacterales is generally mediated by plasmid-encoded AmpC and/or extended-spectrum β-lactamase (ESBL) enzymes, whereas resistance to carbapenems is typically driven by carbapenemases, including OXA-48-like and KPC-like serine β-lactamases, as well as members of the VIM, IMP and NDM families of metallo-β-lactamases (MBLs) (2). Strains carrying these resistance determinants often show cross-resistance to other antimicrobial classes, such as fluoroquinolones, aminoglycosides, and trimethoprim/sulfamethoxazole, due to the co-localization of multiple resistance genes on mobile genetic elements, including integrons, transposons, and plasmids, which are commonly acquired through horizontal gene transfer (3).

Infections caused by MDR Enterobacterales were historically confined to hospital settings. However, the location of AmpC-, ESBL- and carbapenemase-encoding genes on mobile plasmids, together with the association of these genes with epidemic high-risk clones, has facilitated dissemination in the community (4, 5). Hospital admission is therefore often required for administration of intravenous therapy for infections caused by strains producing broad-spectrum β-lactamases, with carbapenems used to treat infections caused by AmpC- and ESBL-producing organisms, and newer β-lactam/β-lactamase inhibitor combinations (such as ceftazidime/avibactam, imipenem/relebactam, meropenem/vaborbactam and aztreonam/avibactam) are used to treat infections caused by carbapenemase producers (6). The reliance on intravenous therapy highlights an important, unmet need for effective oral treatment options that would enable outpatient management, reduce hospital admissions, and mitigate healthcare-associated complications and costs (7–9).

In this context, combining oral cephalosporins with novel oral β-lactamase inhibitors represents an attractive therapeutic strategy. Ceftibuten is a third-generation oral oxyimino-cephalosporin with favorable tolerability and safety profiles and documented clinical efficacy in respiratory and urinary tract infections (10). Notably, ceftibuten exhibits enhanced intrinsic stability against several β-lactamases with cephalosporinase activity, making it a promising partner for β-lactamase inhibitors (11). Based on this rationale, ceftibuten is currently under clinical evaluation in combination with three orally available β-lactamase inhibitor prodrugs: avibactam tomilopil (ARX-1796) (12), ledaborbactam etzadroxil (VNRX-7145) (13), and xeruborbactam isoboxil (QPX7831) (14), which are converted *in vivo* into their active forms. Avibactam and ledaborbactam inhibit class A, C, and D serine β-lactamases (15, 16), while xeruborbactam, a cyclic boronate inhibitor, displays broader activity that includes inhibition of MBLs (17). Accordingly, ceftibuten, in combination with avibactam, ledaborbactam, or xeruborbactam, may represent valuable options for the oral treatment of infections such as urinary tract infections caused by β-lactam-resistant Enterobacterales.

We evaluated the activity of ceftibuten alone and in combination with avibactam, ledaborbactam, or xeruborbactam against recombinant and clinical Enterobacterales isolates producing well-characterized β-lactamases. We also evaluated the activity of ceftazidime, ceftazidime/avibactam and meropenem as comparator agents. MICs were determined using the reference broth microdilution method with CLSI-recommended methodologies and quality control strains (*Escherichia coli* NCTC 13353, *Klebsiella pneumoniae* ATCC BAA2814, *E. coli* ATCC 25922 and *K. pneumoniae* ATCC 700603) (18). β-Lactamase inhibitors were tested at a fixed concentration of 4 mg/L as recommended by CLSI guidelines (18) and for comparative reasons, and susceptibility was interpreted using ceftibuten breakpoints, applying both EUCAST (S ≤ 1 mg/L) and CLSI (S ≤ 16 mg/L) criteria for clinical isolates.

We leveraged our previously engineered library of recombinant *E. coli* TG1 derivatives producing 50 distinct β-lactamase genes cloned into the high-copy number plasmid pUCP24 (19, 20). This collection encompassed β-lactamases from all Ambler classes and was further enriched for the present study with new β-lactamase genes. These newly introduced β-lactamase genes were synthesized by Integrated DNA Technologies (Coralville, IA, USA). The genes included some that encode less prevalent class D enzymes as well as metallo-β-lactamase variants known to harbor amino acid substitutions associated with reduced susceptibility to boronate inhibitors, namely VIM-83, NDM-9, IMP-1, IMP-6, IMP-10, OXA-163, OXA-372, and SIM-1 (21, 22). Comparative MIC data for the recombinant strains collection are presented in Table 1. Analysis of the resulting ceftibuten and ceftazidime MIC values enabled assessment of the intrinsic stability of the cephalosporin alone. Production of most β-lactamases significantly elevated the MICs of both cephalosporins, confirming the robustness of our experimental β-lactamase-producing *E. coli* model. Overall, ceftazidime was more extensively affected across most β-lactamase classes, whereas ceftibuten exhibited greater collective stability, especially against class A enzymes. Enhanced stability of ceftibuten against these enzymes has previously been noted in laboratory strains by Livermore et al. (11) and Le Terrier et al. (23). This observation is of great clinical relevance as the widespread dissemination of ESBLs (such as CTX-M-15) in epidemic strains of Enterobacterales, such as *E. coli* ST131, drives cephalosporin-resistant infections, particularly urinary tract infections, in the community setting (24, 25). Ceftibuten was also scarcely affected by class D enzymes, including extended-spectrum OXA variants and OXA-48, which weakly hydrolyzes cephalosporins. By contrast, ceftibuten was particularly compromised by class C β-lactamases, with most AmpC producers yielding MICs of >64 mg/L, consistent with previous reports. Both ceftazidime and ceftibuten were markedly affected by MBLs, especially NDM variants, resulting in high MICs.

**TABLE 1:** Antibiotic susceptibility data for ceftibuten-based combinations against quality control strains and isogenic *E. coli* TG1 transformants producing the most relevant class A, B, C, and D β-lactamases found in Enterobacterales

Strain	Ambler class	Main phenotype	MIC (mg/L)^*a*^^,^^*b*^						
			CTB (*R* > 1)	CTB/A (*R* > 1)	CTB/L (*R* > 1)	CTB/X (*R* > 1)	CAZ (*R* > 4)	CAZ/A (*R* > 8)	MEM (*R* > 8)
*E. coli* TG1	–	Wild-type	0.25	≤0.06	≤0.06	≤0.06	0.125	0.125	≤0.06
*E. coli* TG1 (pUCP24)	–	Wild-type	0.25	≤0.06	≤0.06	≤0.06	0.125	0.125	≤0.06
*E. coli* ATCC 25922	–	Wild-type	0.25	≤0.06	≤0.06	≤0.06	–	–	≤0.06
*E. coli* NCTC 13353	–	ESBL	32	≤0.06	≤0.06	≤0.06	–	–	–
*K. pneumoniae* ATCC 700603	–	ESBL	–	–	–	–	32	0.5	–
*K. pneumoniae* ATCC BAA-2814	–	Carbapenemase	16	0.125	0.5	0.125	–	–	64
GES-1	A	ESBL	0.125	≤0.06	≤0.06	≤0.06	4	0.125	≤0.06
GES-5	A	Carbapenemase	0.5	≤0.06	≤0.06	≤0.06	0.25	0.125	≤0.06
GES-7	A	ESBL	≤0.06	≤0.06	≤0.06	≤0.06	32	0.25	≤0.06
GES-20	A	Carbapenemase	0.125	≤0.06	≤0.06	≤0.06	0.25	0.125	≤0.06
CTX-M-9	A	ESBL	0.5	≤0.06	0.125	≤0.06	0.5	0.125	≤0.06
CTX-M-15	A	ESBL	16	≤0.06	≤0.06	≤0.06	32	0.125	≤0.06
SHV-12	A	ESBL	8	≤0.06	0.125	≤0.06	>64	0.25	≤0.06
PER-1	A	ESBL	128	0.5	0.25	≤0.06	>64	2	≤0.06
VEB-1	A	ESBL	16	≤0.06	≤0.06	≤0.06	>64	0.25	≤0.06
VEB-25	A	ESBL	2	0.25	≤0.06	≤0.06	>64	64	≤0.06
BEL-1	A	ESBL	0.5	≤0.06	≤0.06	≤0.06	4	0.125	≤0.06
SFO-1	A	ESBL	1	≤0.06	≤0.06	≤0.06	8	0.25	≤0.06
KPC-2	A	Carbapenemase	2	≤0.06	≤0.06	≤0.06	16	0.25	1
KPC-35	A	ESBL	2	≤0.06	≤0.06	≤0.06	>64	8	≤0.06
KPC-3	A	Carbapenemase	1	≤0.06	≤0.06	≤0.06	32	0.25	1
KPC-31	A	ESBL	1	≤0.06	≤0.06	≤0.06	64	16	≤0.06
VIM-1	B	Carbapenemase	64	64	64	1	>64	>64	0.5
VIM-2	B	Carbapenemase	2	2	0.5	≤0.06	1	1	≤0.06
VIM-4	B	Carbapenemase	32	32	4	≤0.06	8	4	0.125
VIM-20	B	Carbapenemase	8	4	1	≤0.06	2	1	≤0.06
VIM-83	B	Carbapenemase	128	128	64	0.25	>64	>64	0.5
IMP-1	B	Carbapenemase	32	16	16	0.25	>64	>64	≤0.06
IMP-6	B	Carbapenemase	32	16	16	16	32	16	4
IMP-8	B	Carbapenemase	128	128	128	8	>64	>64	0.25
IMP-10	B	Carbapenemase	32	16	16	8	>64	>64	1
IMP-13	B	Carbapenemase	64	64	64	4	>64	>64	≤0.06
IMP-23	B	Carbapenemase	16	16	16	8	>64	>64	0.25
IMP-94	B	Carbapenemase	16	16	16	0.25	>64	>64	≤0.06
NDM-1	B	Carbapenemase	512	512	512	16	>64	>64	2
NDM-5	B	Carbapenemase	512	512	512	64	>64	>64	32
NDM-7	B	Carbapenemase	512	512	512	32	>64	>64	16
NDM-9	B	Carbapenemase	>512	>512	>512	64	>64	>64	16
NDM-23	B	Carbapenemase	512	512	512	16	>64	>64	2
SPM-1	B	Carbapenemase	64	32	32	32	>64	64	0.5
SIM-1	B	Carbapenemase	64	64	64	8	64	32	0.5
AmpC (*Klebsiella aerogenes*)	C	AmpC	512	2	2	0.5	64	0.25	≤0.06
AmpC (*Enterobacter cloacae*)	C	AmpC	512	1	0.5	0.25	32	0.125	≤0.06
CMH-7	C	AmpC	256	2	0.25	0.125	32	0.125	≤0.06
CMY-2	C	AmpC	>512	4	1	1	>64	0.5	≤0.06
DHA-1	C	AmpC	64	0.125	1	≤0.06	32	0.125	≤0.06
FOX-4	C	AmpC	8	0.125	0.125	≤0.06	>64	1	≤0.06
FOX-14	C	AmpC	256	4	0.25	0.125	>64	16	0.125
OXA-1	D	Narrow-spectrum oxacillinase	0.25	≤0.06	≤0.06	≤0.06	0.125	0.125	≤0.06
OXA-2	D	Narrow-spectrum oxacillinase	0.25	≤0.06	≤0.06	≤0.06	2	≤0.06	≤0.06
OXA-10	D	Narrow-spectrum oxacillinase	0.25	≤0.06	≤0.06	≤0.06	0.125	≤0.06	≤0.06
OXA-14	D	ESBL	0.25	≤0.06	≤0.06	≤0.06	32	4	≤0.06
OXA-15	D	ESBL	0.25	≤0.06	≤0.06	≤0.06	32	2	≤0.06
OXA-48	D	Carbapenemase	0.25	≤0.06	≤0.06	≤0.06	0.25	≤0.06	0.5
OXA-163	D	ESBL	1	≤0.06	≤0.06	≤0.06	8	≤0.06	≤0.06
OXA-372	D	Carbapenemase	0.125	≤0.06	0.125	≤0.06	0.5	≤0.06	≤0.06

^*a*^ CTB, ceftibuten; CTB/A, ceftibuten/avibactam; CTB/L, ceftibuten/ledaborbactam; CTB/X, ceftibuten/xeruborbactam; CAZ, ceftazidime; CAZ/A, ceftazidime/avibactam; MEM, meropenem.

^*b*^ Following EUCAST (v15.0) breakpoints for Enterobacterales.

Combining ceftibuten with avibactam or ledaborbactam strongly reduced the MIC values for transformants producing class A, C, and D β-lactamases, frequently restoring susceptibility to near wild-type levels. No substantial differences between avibactam and ledaborbactam were observed in this setting. These findings corroborate those of prior studies with ceftibuten/avibactam (23) and provide the first systematic demonstration of ledaborbactam activity against a broad isogenic collection of β-lactamase-producing Enterobacterales. Xeruborbactam similarly restored ceftibuten activity against transformants producing class A, C, and D enzymes. Importantly, it also restored susceptibility against VIM-type MBL producers. By contrast, xeruborbactam did not restore ceftibuten activity against some IMP-producing transformants and none of NDM-producing transformants. This observation can be explained by two main factors. First, IMP enzymes are among the least effectively inhibited by cyclic boronates, as also observed with taniborbactam, yielding IC_50_ values exceeding 500 nM. These values are markedly higher than those reported for VIM (14 nM) or NDM (55 nM) (15). This study also included recently described IMP variants associated with enhanced resistance to xeruborbactam, such as IMP-6, IMP-10, and IMP-23, which harbor substitutions like S226G and V67F. These variants have previously shown negligible reductions in β-lactam MICs upon addition of xeruborbactam (22, 26). Consistently, biochemical characterization of IMP-10 demonstrated reduced susceptibility, with an IC_50_ of 73 µM compared with 2.7 µM for IMP-1 (21). Second, although NDM enzymes are more effectively inhibited by xeruborbactam than IMPs, they confer very high baseline ceftibuten MICs due to their strong catalytic activity against cephalosporins (27). Consequently, even potent inhibition of NDM enzymes is insufficient to reduce ceftibuten MICs to values compatible with susceptibility.

To validate these findings in a clinically relevant context, we evaluated the activity of three ceftibuten combinations against 300 non-duplicated, whole-genome-sequenced clinical carbapenemase-producing Enterobacterales (CPE) isolates collected from 39 Spanish hospitals between 2017 and 2024, including 100 OXA-48-like, 100 KPC-like, and 100 MBL producers (Table 2). The characteristics of this collection are described in our recent work (20), as well as in Tables S1 and S2. Detailed MIC values for ceftibuten combinations and comparators, as well as phenotypic and genomic data of this collection, are also provided in Tables S3 to S5. Ceftibuten/xeruborbactam was the most active combination across the whole collection, achieving susceptibility rates of 75.7% and 94.7% according to EUCAST and CLSI criteria, respectively, accompanied by marked reductions in MIC_50_ and MIC_90_ values. By contrast, ceftibuten/avibactam and ceftibuten/ledaborbactam showed lower overall susceptibility rates, reflecting their lack of activity against MBL-producing isolates. This global overview highlights ceftibuten/xeruborbactam as the ceftibuten combination with the broadest activity against CPE. These findings could be complemented by future studies evaluating these combinations against collections of AmpC- or ESBL-producing Enterobacterales. These studies should also include urinary isolates and comparator agents commonly used to treat urinary tract infections, such as ciprofloxacin, trimethoprim-sulfamethoxazole, or fosfomycin.

**TABLE 2:** *In vitro* antibiotic susceptibility activity data of ceftibuten in combination with avibactam, ledaborbactam and xeruborbactam and comparator agents against clinical CPE isolates with defined carbapenemase content

Carbapenemase type	Antimicrobial agent(s)^*a*^	MIC (mg/L)												MIC_50_	MIC_90_	Range	EUCAST*^b^*			CLSI*^c^*		
		≤0.06	0.125	0.25	0.5	1	2	4	8	16	32	64	>64				% S	% I	% R	% S	% I	% R
All (*n* = 300)	CTB	15 (5.0%)	25 (8.3%)	29 (9.7%)	35 (11.7%)	40 (13.3%)	51 (17.0%)	66 (22.0%)	96 (32.0%)	135 (45.0%)	167 (55.7%)	199 (66.3%)	300 (100.0%)	32	>64	≤0.06 - >64	13.3	–	86.7	32.0	13.0	55.0
	CTB/A	90 (30.0%)	130 (43.3%)	168 (56.0%)	182 (60.7%)	192 (64.0%)	194 (64.7%)	200 (66.7%)	202 (67.3%)	206 (68.7%)	226 (75.3%)	259 (86.3%)	300 (100.0%)	0.25	>64	≤0.06 - >64	64.0	–	36.0	67.3	1.4	31.3
	CTB/L	72 (24.0%)	116 (38.7%)	154 (51.3%)	175 (58.3%)	189 (63.0%)	198 (66.0%)	203 (67.7%)	211 (70.3%)	228 (76.0%)	245 (81.7%)	270 (90.0%)	300 (100.0%)	0.25	64	≤0.06 - >64	63.0	–	37.0	70.3	5.7	24.0
	CTB/X	99 (33.0%)	139 (46.3%)	183 (61.0%)	211 (70.3%)	227 (75.7%)	254 (84.7%)	272 (90.7%)	284 (94.7%)	290 (96.7%)	292 (97.3%)	295 (98.3%)	300 (100.0%)	0.25	4	≤0.06 - 64	75.7	–	24.3	94.7	2.0	3.3
	CAZ	2 (0.7%)	5 (1.7%)	11 (3.7%)	18 (6.0%)	27 (9.0%)	34 (11.3%)	40 (13.3%)	45 (15.0%)	54 (18.0%)	66 (22.0%)	83 (27.7%)	300 (100.0%)	>64	>64	≤0.06 - >64	9.0	6.0	85.0	13.3	1.7	85.0
	CAZ/A	8 (2.7%)	24 (8.0%)	37 (12.3%)	77 (25.7%)	121 (40.3%)	155 (51.7%)	172 (57.3%)	187 (62.3%)	195 (65.0%)	201 (67.0%)	205 (68.3%)	300 (100.0%)	2	>64	≤0.06 - >64	62.3	–	37.7	62.3	–	37.7
	MEM	6 (2.0%)	14 (4.7%)	44 (14.7%)	90 (30.0%)	137 (45.7%)	172 (57.3%)	198 (66.0%)	222 (74.0%)	248 (82.7%)	261 (87.0%)	278 (92.7%)	300 (100.0%)	2	64	≤0.06 - >64	57.3	25.4	17.3	45.7	11.6	42.7
OXA-48-like (*n* = 100)	CTB	15 (15.0%)	25 (25.0%)	28 (28.0%)	32 (32.0%)	35 (35.0%)	39 (39.0%)	41 (41.0%)	47 (47.0%)	58 (58.0%)	66 (66.0%)	70 (70.0%)	100 (100.0%)	16	>64	≤0.06 - 64	35.0	–	65.0	47.0	11.0	42.0
	CTB/A	42 (42.0%)	66 (66.0%)	82 (82.0%)	92 (92.0%)	96 (96.0%)	96 (96.0%)	96 (96.0%)	96 (96.0%)	97 (97.0%)	97 (97.0%)	98 (98.0%)	100 (100.0%)	0.125	0.5	≤0.06 - >64	96.0	–	4.0	96.0	1.0	3.0
	CTB/L	35 (35.0%)	55 (55.0%)	70 (70.0%)	80 (80.0%)	91 (91.0%)	95 (95.0%)	96 (96.0%)	97 (97.0%)	97 (97.0%)	97 (97.0%)	98 (98.0%)	100 (100.0%)	0.125	1	≤0.06 - >64	91.0	–	9.0	97.0	0.0	3.0
	CTB/X	41 (41.0%)	61 (61.0%)	74 (74.0%)	87 (87.0%)	94 (94.0%)	94 (94.0%)	95 (95.0%)	96 (96.0%)	97 (97.0%)	98 (98.0%)	99 (99.0%)	100 (100.0%)	0.125	1	≤0.06 - >64	94.0	–	6.0	96.0	1.0	3.0
	CAZ	2 (2.0%)	5 (5.0%)	10 (10.0%)	17 (17.0%)	26 (26.0%)	32 (32.0%)	36 (36.0%)	36 (36.0%)	36 (36.0%)	41 (41.0%)	46 (46.0%)	100 (100.0%)	>64	>64	≤0.06 - > 64	26.0	10.0	64.0	36.0	0.0	64.0
	CAZ/A	7 (7.0%)	18 (18.0%)	28 (28.0%)	53 (53.0%)	77 (77.0%)	90 (90.0%)	97 (97.0%)	97 (97.0%)	98 (98.0%)	99 (99.0%)	99 (99.0%)	100 (100.0%)	0.5	2	≤0.06 - >64	97.0	–	3.0	97.0	–	3.0
	MEM	2 (2.0%)	6 (6.0%)	20 (20.0%)	43 (43.0%)	65 (65.0%)	74 (74.0%)	75 (75.0%)	78 (78.0%)	89 (89.0%)	94 (94.0%)	100 (100.0%)		1	32	≤0.06 - 64	74.0	15.0	11.0	65.0	9.0	26.0
KPC-like (*n* = 100)	CTB			1 (1.0%)	3 (3.0%)	5 (5.0%)	12 (12.0%)	22 (22.0%)	44 (44.0%)	68 (68.0%)	86 (86.0%)	91 (91.0%)	100 (100.0%)	16	64	0.25 - >64	5.0	–	95.0	44.0	24.0	32.0
	CTB/A	48 (48.0%)	64 (64.0%)	86 (86.0%)	90 (90.0%)	96 (96.0%)	97 (97.0%)	98 (98.0%)	99 (99.0%)	99 (99.0%)	100 (100.0%)			0.125	0.5	≤0.06 - 32	96.0	–	4.0	99.0	0.0	1.0
	CTB/L	37 (37.0%)	61 (61.0%)	84 (84.0%)	95 (95.0%)	97 (97.0%)	99 (99.0%)	99 (99.0%)	99 (99.0%)	99 (99.0%)	99 (99.0%)	100 (100.0%)		0.125	0.5	≤0.06 - 64	97.0	–	3.0	99.0	0.0	1.0
	CTB/X	50 (50.0%)	68 (68.0%)	90 (90.0%)	94 (94.0%)	95 (95.0%)	99 (99.0%)	99 (99.0%)	99 (99.0%)	99 (99.0%)	99 (99.0%)	99 (99.0%)	100 (100.0%)	≤0.06	0.25	≤0.06 - >64	95.0	–	5.0	99.0	0.0	1.0
	CAZ			1 (1.0%)	1 (1.0%)	1 (1.0%)	2 (2.0%)	4 (4.0%)	9 (9.0%)	16 (16.0%)	23 (23.0%)	32 (32.0%)	100 (100.0%)	>64	>64	0.25 - >64	1.0	8.0	91.0	4.0	5.0	91.0
	CAZ/A	1 (1.0%)	6 (6.0%)	9 (9.0%)	24 (24.0%)	44 (44.0%)	65 (65.0%)	75 (75.0%)	89 (89.0%)	95 (95.0%)	98 (98.0%)	98 (98.0%)	100 (100.0%)	2	16	≤0.06 - >64	89.0	–	11.0	89.0	–	11.0
	MEM	2 (2.0%)	3 (3.0%)	6 (6.0%)	8 (8.0%)	13 (13.0%)	32 (32.0%)	45 (45.0%)	55 (55.0%)	66 (66.0%)	70 (70.0%)	80 (80.0%)	100 (100.0%)	8	>64	≤0.06 - >64	32.0	34.0	34.0	13.0	19.0	68.0
MBL (*n* = 100)	CTB							3 (3.0%)	5 (5.0%)	9 (9.0%)	15 (15.0%)	38 (38.0%)	100 (100.0%)	>64	>64	4 - >64	0.0	–	100.0	5.0	4.0	91.0
	CTB/A						1 (1.0%)	6 (6.0%)	7 (7.0%)	10 (10.0%)	29 (29.0%)	61 (61.0%)	100 (100.0%)	64	>64	2 - >64	0.0	–	100.0	7.0	3.0	90.0
	CTB/L					1 (1.0%)	4 (4.0%)	8 (8.0%)	15 (15.0%)	32 (32.0%)	49 (49.0%)	72 (72.0%)	100 (100.0%)	64	>64	1 - >64	1.0	–	99.0	15.0	17.0	68.0
	CTB/X	9 (9.0%)	11 (11.0%)	19 (19.0%)	30 (30.0%)	38 (38.0%)	61 (61.0%)	78 (78.0%)	89 (89.0%)	94 (94.0%)	95 (95.0%)	97 (97.0%)	100 (100.0%)	2	16	≤0.06 - >64	38.0	–	62.0	89.0	5.0	6.0
	CAZ									2 (2.0%)	2 (2.0%)	5 (5.0%)	100 (100.0%)	>64	>64	16 - >64	0.0	0.0	100.0	0.0	0.0	100.0
	CAZ/A								1 (1.0%)	2 (2.0%)	4 (4.0%)	8 (8.0%)	100 (100.0%)	>64	>64	8 - >64	1.0	–	99.0	1.0	–	99.0
	MEM	2 (2.0%)	5 (5.0%)	18 (18.0%)	39 (39.0%)	59 (59.0%)	66 (66.0%)	78 (78.0%)	89 (89.0%)	93 (93.0%)	97 (97.0%)	98 (98.0%)	100 (100.0%)	1	16	≤0.06 - >64	66.0	27.0	7.0	59.0	7.0	34.0
VIM (*n* = 63)	CTB							2 (3.2%)	4 (6.3%)	8 (12.7%)	13 (20.6%)	30 (47.6%)	63 (100.0%)	>64	>64	4 - >64	0.0	–	100.0	6.3	6.4	87.3
	CTB/A						1 (1.6%)	5 (7.9%)	6 (9.5%)	9 (14.3%)	27 (42.9%)	54 (85.7%)	63 (100.0%)	64	>64	2 - >64	0.0	–	100.0	9.5	4.8	85.7
	CTB/L					1 (1.6%)	3 (4.8%)	7 (11.1%)	14 (22.2%)	31 (49.2%)	45 (71.4%)	60 (95.2%)	63 (100.0%)	32	64	1 - > 64	1.6	–	98.4	22.2	27.0	50.8
	CTB/X	9 (14.3%)	11 (17.5%)	18 (28.6%)	29 (46.0%)	37 (58.7%)	51 (81.0%)	60 (95.2%)	63 (100.0%)					1	4	≤0.06 - 8	58.7	–	41.3	100.0	0.0	0.0
	CAZ									2 (3.2%)	2 (3.2%)	5 (7.9%)	63 (100.0%)	>64	>64	16 - >64	0.0	0.0	100.0	0.0	0.0	100.0
	CAZ/A								1 (1.6%)	2 (3.2%)	4 (6.3%)	8 (12.7%)	63 (100.0%)	>64	>64	8 - >64	1.6	–	98.4	1.6	–	98.4
	MEM	2 (3.2%)	5 (7.9%)	17 (27.0%)	36 (57.1%)	52 (82.5%)	56 (88.9%)	57 (90.5%)	61 (96.8%)	61 (96.8%)	62 (98.4%)	62 (98.4%)	63 (100.0%)	0.5	4	≤0.06 - >64	88.9	7.9	3.2	82.5	6.4	11.1
NDM (*n* = 32)	CTB							1 (3.1%)	1 (3.1%)	1 (3.1%)	1 (3.1%)	6 (18.8%)	32 (100.0%)	>64	>64	4 - >64	0.0	–	100.0	3.1	0.0	96.9
	CTB/A							1 (3.1%)	1 (3.1%)	1 (3.1%)	1 (3.1%)	4 (12.5%)	32 (100.0%)	>64	>64	4 - >64	0.0	–	100.0	3.1	0.0	96.9
	CTB/L						1 (3.1%)	1 (3.1%)	1 (3.1%)	1 (3.1%)	2 (6.3%)	9 (28.1%)	32 (100.0%)	>64	>64	2 - >64	0.0	-	100.0	3.1	0.0	96.9
	CTB/X			1 (3.1%)	1 (3.1%)	1 (3.1%)	9 (28.1%)	16 (50.0%)	22 (68.8%)	26 (81.3%)	27 (84.4%)	29 (90.6%)	32 (100.0%)	4	64	0.25 - >64	3.1	–	96.9	68.8	12.5	18.7
	CAZ												32 (100.0%)	>64	>64	>64 - >64	0.0	100.0	0.0	0.0	0.0	100.0
	CAZ/A												32 (100.0%)	>64	>64	>64 - >64	0.0	–	100.0	0.0	–	100.0
	MEM					3 (9.4%)	5 (15.6%)	16 (50.0%)	23 (71.9%)	27 (84.4%)	30 (93.8%)	31 (96.9%)	32 (100.0%)	4	32	1 - >64	15.6	68.8	15.6	9.4	6.2	84.4

^*a*^ CTB, ceftibuten; CTB/A, ceftibuten/avibactam; CTB/L, ceftibuten/ledaborbactam; CTB/X, ceftibuten/xeruborbactam; CAZ, ceftazidime; CAZ/A, ceftazidime/avibactam; MEM, meropenem.

^*b*^ Following EUCAST (v15.0) breakpoints for Enterobacterales.

^*c*^ Following CLSI M100 (2025) breakpoints for Enterobacterales.

When analyzed by carbapenemase type, all three inhibitors strongly potentiated ceftibuten activity against OXA-48-like and KPC-producing isolates, reducing MIC_50_ and MIC_90_ by 128- to >256-fold and by 64- to 256-fold for MIC_90_ values. Susceptibility rates against these serine carbapenemase producers were close to 95%–99% for all combinations, regardless of whether EUCAST or CLSI breakpoints were applied, consistent with previously published data for international collections (28–30). Among MBL producers, only ceftibuten/xeruborbactam retained relevant activity. Susceptibility rates varied substantially, depending on the breakpoint applied, increasing from 38% with EUCAST criteria to 89% with CLSI breakpoints. This breakpoint dependency was particularly evident among VIM producers, for which all isolates were inhibited at ≤8 mg/L, resulting in 100% susceptibility using CLSI criteria. On the other hand, activity against NDM producers was more limited, although very important breakpoint-dependent increases in susceptibility were also noted, with a susceptibility rate of 3.1% according to EUCAST breakpoints and of 68.8% according to CLSI breakpoints. The higher activity of ceftibuten/xeruborbactam against VIM-type enzymes compared with NDM producers aligns with recent reports and underscores the persistent challenge posed by NDM-mediated resistance, even with advanced MBL inhibitors (31). Overall, these findings provide a robust *in vitro* foundation supporting the clinical development of oral ceftibuten combinations, particularly against serine carbapenemase-producing Enterobacterales, while anticipating some limitations against MBL-producing organisms, particularly NDM-producing strains. We also acknowledge substantial challenges in interpreting and reporting susceptibility data for ceftibuten/xeruborbactam against MBL-producing strains, as the MICs of many isolates often fall within the 1–16 mg/L range separating EUCAST and CLSI breakpoints. These differences in breakpoints are consistent with the frameworks defined by EUCAST and CLSI (32, 33), which integrate PK/PD analyses with microbiological and clinical data to different degrees. Consequently, EUCAST often yields more conservative thresholds than the less restrictive criteria of CLSI, which may result in clinically relevant variability in the perceived eligibility of isolates for oral therapy. As noted above, this is particularly relevant in our data set regarding the potential clinical use of ceftibuten/xeruborbactam against MBL-producing isolates, where breakpoint-dependent categorization could significantly influence treatment decisions.

## SUPPLEMENTAL MATERIAL

Supplemental material aac.00195-26-s0001.docx Tables S1 to S5.

ASM does not own the copyrights to Supplemental Material that may be linked to, or accessed through, an article. The authors have granted ASM a non-exclusive, world-wide license to publish the Supplemental Material files. Please contact the corresponding author directly for reuse.
