# Supplementary material for: Comparative activity of ceftibuten combinations with avibactam, ledaborbactam, and xeruborbactam against recombinant β-lactamase-producing Escherichia coli and contemporary WGS-characterized strains of carbapenemase-producing Enterobacterales
Source: Antimicrob Agents Chemother. 2026 May 29;70(7):e00195-26. doi: 10.1128/aac.00195-26 (PMC13321838; doi:10.1128/aac.00195-26)
Supplement: Supplemental material — Tables S1 to S5. [file aac.00195-26-s0001.docx]

| **Table S1.** Distribution of the isolates studied according to their species. | | | | |
| --- | --- | --- | --- | --- |
| **Bacterial species** | **All** | **OXA-48-like** | **KPC-like** | **MBL** |
|  | **(n=300)** | **(n=100)** | **(n=100)** | **(n=100)** |
| *Citrobacter freundii* | 16 | 4 | 7 | 5 |
| *Enterobacter cloacae complex* | 55 | 18 | 8 | 29 |
| *Escherichia coli* | 40 | 17 | 9 | 14 |
| *Klebsiella aerogenes* | 2 | - | - | 2 |
| *Klebsiella oxytoca* | 19 | 5 | 4 | 10 |
| *Klebsiella pneumoniae* | 166 | 56 | 72 | 38 |
| *Providencia rettgeri* | 1 | - | - | 1 |
| *Raoultella planticola* | 1 | - | - | 1 |

| **Table S2.** Distribution of isolates according to the type of carbapenemase produced. | | | | | | | | |
| --- | --- | --- | --- | --- | --- | --- | --- | --- |
| **OXA-48-like (n=100)** | |  | **KPC-like (n=100)** | | |  | **MBL (n=100)** | |
| OXA-48 | 96 |  | KPC-2 | | 42 |  | IMP-8 | 4 |
| OXA-181 | 2 |  | KPC-3 | | 54 |  | IMP-22 | 1 |
| OXA-244 | 2 |  | KPC-23 | | 1 |  | NDM-1 | 20 |
|  |  |  | KPC-31 | | 1 |  | NDM-5 | 6 |
|  |  |  | KPC-66 | | 1 |  | NDM-7 | 4 |
|  |  |  | KPC-132 | | 1 |  | NDM-14 | 1 |
|  |  |  |  | |  |  | NDM-23 | 1 |
|  |  |  |  | |  |  | VIM-1 | 61 |
|  |  | |  |  |  |  | VIM-4 | 1 |
|  |  | |  |  |  |  | VIM-23 | 1 |

| **Table S3.** Antimicrobial susceptibility data for ceftibuten combinations and comparator agents against OXA-48-like-producing Enterobacterales (n=100). | | | | | | | | | | | | |
| --- | --- | --- | --- | --- | --- | --- | --- | --- | --- | --- | --- | --- |
| **BioProject ID** | **Genome number** | **Species** | **MLST** | **Hospital code** | **Carbapenemase** | **MIC (mg/L)^a^** | | | | | | |
|  |  |  |  |  |  | **CTB**  **(R>1)** | **CTB/A**  **(R>1)** | **CTB/L**  **(R>1)** | **CTB/X**  **(R>1)** | **CAZ**  **(R>4)** | **CAZ/A**  **(R>8)** | **MEM**  **(R>8)** |
| PRJEB42440 | AI2595 | *Klebsiella pneumoniae* | 405 | MAD01 | OXA-48 | >64 | 0.125 | 2 | 0.5 | >64 | 4 | 16 |
| PRJEB42440 | AI2600 | *Klebsiella pneumoniae* | 405 | MAD01 | OXA-48 | >64 | 0.125 | 0.25 | 0.125 | >64 | 4 | 16 |
| PRJEB42440 | AI2604 | *Klebsiella pneumoniae* | 11 | MAD01 | OXA-48 | 16 | 0.125 | 0.125 | 0.125 | >64 | 4 | 0.25 |
| PRJEB42440 | AI2838 | *Klebsiella pneumoniae* | 307 | CAT01 | OXA-48 | >64 | 0.25 | 4 | 0.25 | >64 | 1 | 64 |
| PRJEB42440 | AH0326 | *Klebsiella pneumoniae* | 392 | CAT01 | OXA-48 | 16 | 0.125 | 0.25 | 0.125 | 1 | 1 | 16 |
| PRJEB42440 | AI2847 | *Klebsiella pneumoniae* | 307 | CAT01 | OXA-48 | >64 | 0.25 | 0.25 | 0.5 | >64 | 2 | 64 |
| PRJEB42440 | AH0327 | *Klebsiella pneumoniae* | 392 | CAT01 | OXA-48 | 16 | ≤0.06 | 0.125 | ≤0.06 | >64 | 1 | 1 |
| PRJEB42440 | AI2849 | *Klebsiella pneumoniae* | 147 | CAT02 | OXA-48 | >64 | 0.5 | 2 | 1 | >64 | 1 | 16 |
| PRJEB42440 | AI2852 | *Escherichia coli* | 10 | CAT02 | OXA-48 | 4 | 0.25 | 0.25 | 0.25 | >64 | 1 | 32 |
| PRJEB39112 | AI2855 | *Klebsiella pneumoniae* | 147 | CAT02 | OXA-48 | >64 | 0.5 | 0.25 | 1 | >64 | 4 | 32 |
| PRJEB39112 | AI2856 | *Klebsiella pneumoniae* | 147 | CAT02 | OXA-48 | >64 | 0.25 | 1 | 0.5 | >64 | 1 | 16 |
| PRJEB42440 | AI2866 | *Enterobacter cloacae complex* | 90 | MAD02 | OXA-48 | 0.5 | ≤0.06 | ≤0.06 | ≤0.06 | 4 | 1 | 1 |
| PRJEB42440 | AI2870 | *Klebsiella pneumoniae* | 11 | MAD03 | OXA-48 | >64 | 0.5 | 0.5 | 0.5 | >64 | 2 | 8 |
| PRJEB42440 | AI2879 | *Klebsiella pneumoniae* | 11 | MAD03 | OXA-48 | 16 | ≤0.06 | 0.125 | 0.125 | >64 | >64 | 1 |
| PRJEB42440 | AI2882 | *Klebsiella pneumoniae* | 307 | MAD03 | OXA-48 | ≤0.06 | ≤0.06 | 0.125 | ≤0.06 | >64 | 1 | 2 |
| PRJEB53700 | 20220622 | *Escherichia coli* | 10 | AND16 | OXA-244 | >64 | ≤0.06 | 0.5 | 0.125 | 32 | 0.25 | ≤0.06 |
| PRJEB42440 | AI2890 | *Escherichia coli* | 538 | MAD03 | OXA-48 | 0.5 | 0.125 | ≤0.06 | ≤0.06 | 2 | 0.5 | 2 |
| PRJEB42440 | AI2893 | *Klebsiella pneumoniae* | 45 | MAD03 | OXA-48 | ≤0.06 | ≤0.06 | ≤0.06 | ≤0.06 | 0.5 | 0.125 | 0.25 |
| PRJEB42440 | AI2894 | *Escherichia coli* | 58 | MAD03 | OXA-48 | 0.125 | ≤0.06 | ≤0.06 | ≤0.06 | 0.5 | 0.125 | 0.25 |
| PRJEB42440 | AH0329 | *Klebsiella pneumoniae* | 392 | MAD04 | OXA-48 | 0.125 | ≤0.06 | ≤0.06 | ≤0.06 | >64 | 1 | 0.25 |
| PRJEB42440 | AI2926 | *Klebsiella pneumoniae* | 11 | MAD04 | OXA-48 | 64 | 0.125 | 0.125 | 0.125 | >64 | 0.5 | 0.5 |
| PRJEB42440 | AI2938 | *Escherichia coli* | 127 | BAL01 | OXA-48 | 1 | ≤0.06 | 0.125 | ≤0.06 | 32 | 0.25 | 0.5 |
| PRJEB42440 | AI2697 | *Enterobacter cloacae* | 171 | AST01 | OXA-48 | 16 | ≤0.06 | ≤0.06 | 0.125 | >64 | 0.5 | 0.5 |
| PRJEB42440 | AI2661 | *Citrobacter freundii* | 22 | AST01 | OXA-48 | >64 | 1 | 2 | 1 | >64 | 0.25 | 0.5 |
| PRJEB42440 | AI2662 | *Enterobacter cloacae complex* | 171 | AST01 | OXA-48 | >64 | 0.5 | 1 | 16 | >64 | 0.5 | 64 |
| PRJEB42440 | AI2666 | *Citrobacter freundii* | 18 | AST01 | OXA-48 | 64 | 0.5 | 0.5 | 0.5 | >64 | 0.5 | 0.5 |
| PRJEB42440 | AI2679 | *Enterobacter cloacae complex* | 23 | AST01 | OXA-48 | 8 | 0.25 | 0.125 | 0.25 | 2 | 0.5 | 1 |
| PRJEB42440 | AI2683 | *Enterobacter cloacae complex* | 66 | AST01 | OXA-48 | 32 | 0.125 | 0.25 | 0.25 | >64 | 2 | 0.5 |
| PRJEB42440 | AI2686 | *Klebsiella pneumoniae* | 326 | AST01 | OXA-48 | 32 | 0.125 | 0.125 | 0.125 | >64 | 0.5 | 2 |
| PRJEB42440 | AH0330 | *Klebsiella pneumoniae* | 15 | AST01 | OXA-48 | 8 | ≤0.06 | ≤0.06 | ≤0.06 | >64 | 0.5 | 0.5 |
| PRJEB42440 | AI2951 | *Escherichia coli* | 131 | AST01 | OXA-48 | 0.5 | ≤0.06 | ≤0.06 | ≤0.06 | 0.5 | 0.25 | 0.125 |
| PRJEB42440 | AI2693 | *Klebsiella pneumoniae* | 567 | AST01 | OXA-48 | ≤0.06 | ≤0.06 | ≤0.06 | ≤0.06 | 0.125 | ≤0.06 | 0.25 |
| PRJEB42440 | AI2695 | *Enterobacter cloacae complex* | 78 | AST01 | OXA-48 | 4 | ≤0.06 | ≤0.06 | ≤0.06 | 4 | 0.5 | 1 |
| PRJEB42440 | AI2952 | *Klebsiella pneumoniae* | 326 | AST01 | OXA-48 | ≤0.06 | ≤0.06 | ≤0.06 | ≤0.06 | 0.25 | 0.125 | 0.5 |
| PRJEB42440 | AI2959 | *Escherichia coli* | 68 | CLM01 | OXA-48 | 1 | 0.125 | 0.25 | 0.25 | 2 | 1 | 0.5 |
| PRJEB42440 | AI2962 | *Klebsiella pneumoniae* | 307 | CLM01 | OXA-48 | >64 | 0.125 | 0.25 | 0.25 | >64 | 0.5 | 64 |
| PRJEB42440 | AI2966 | *Klebsiella pneumoniae* | 307 | CLM01 | OXA-48 | 8 | ≤0.06 | ≤0.06 | ≤0.06 | >64 | 32 | 1 |
| PRJEB42440 | AI2967 | *Klebsiella pneumoniae* | 405 | CLM01 | OXA-48 | 0.125 | 0.125 | 0.25 | 0.125 | >64 | 0.5 | 1 |
| PRJEB42440 | AH0331 | *Klebsiella pneumoniae* | 104 | CLM01 | OXA-48 | 0.5 | 0.25 | 1 | 0.5 | 1 | 0.5 | 32 |
| PRJEB42440 | AI2908 | *Klebsiella pneumoniae* | 39 | MAD04 | OXA-48 | >64 | 0.125 | 1 | 0.25 | >64 | 2 | 0.5 |
| PRJEB39112 | AI2766 | *Klebsiella pneumoniae* | 147 | CAT03 | OXA-48 | >64 | 0.5 | 1 | 8 | >64 | 2 | 64 |
| PRJEB39112 | AI2767 | *Klebsiella pneumoniae* | 147 | CAT03 | OXA-48 | >64 | 0.25 | 0.5 | 0.25 | >64 | 1 | 16 |
| PRJEB39112 | AI2993 | *Klebsiella pneumoniae* | 147 | CAT03 | OXA-48 | >64 | 0.25 | 0.5 | 0.25 | >64 | 2 | 16 |
| PRJEB39112 | AI2768 | *Klebsiella pneumoniae* | 147 | CAT03 | OXA-48 | >64 | 0.25 | 1 | 0.5 | >64 | 0.5 | 16 |
| PRJEB39112 | AI2996 | *Klebsiella pneumoniae* | 147 | CAT03 | OXA-48 | >64 | 0.5 | 0.5 | 0.5 | >64 | 2 | 16 |
| PRJEB39112 | AI2998 | *Klebsiella pneumoniae* | 147 | CAT03 | OXA-48 | >64 | 0.25 | 0.5 | 0.5 | >64 | 1 | 32 |
| PRJEB42440 | AN2336 | *Klebsiella oxytoca* | 27 | AST01 | OXA-48 | ≤0.06 | ≤0.06 | ≤0.06 | ≤0.06 | 2 | 0.5 | 0.5 |
| PRJEB42440 | AI2787 | *Klebsiella pneumoniae* | 307 | VAL01 | OXA-48 | 16 | ≤0.06 | 0.125 | ≤0.06 | >64 | 1 | 1 |
| PRJEB42440 | AI3011 | *Klebsiella pneumoniae* | 13 | CAT04 | OXA-48 | 0.25 | 0.125 | 0.125 | ≤0.06 | 2 | 1 | 8 |
| PRJEB42440 | AI2801 | *Klebsiella pneumoniae* | 101 | CAT04 | OXA-48 | >64 | 0.5 | 1 | 0.25 | >64 | 4 | 32 |
| PRJEB42440 | AI2935 | *Citrobacter freundii* | 112 | AND02 | OXA-48 | 32 | 0.125 | 0.125 | ≤0.06 | 64 | 0.5 | 1 |
| PRJEB42440 | AI2624 | *Enterobacter cloacae complex* | 114 | VAL02 | OXA-48 | >64 | 64 | 64 | 32 | >64 | 2 | 16 |
| PRJEB42440 | AI2915 | *Enterobacter cloacae complex* | 114 | MAD04 | OXA-48 | >64 | 1 | 1 | 1 | >64 | 4 | 64 |
| PRJEB42440 | AI2949 | *Escherichia coli* | 131 | AST01 | OXA-48 | >64 | 1 | 0.5 | 0.5 | 1 | 0.125 | 0.5 |
| PRJEB42440 | AI2623 | *Klebsiella oxytoca* | 145 | VAL02 | OXA-48 | 32 | 0.25 | 0.125 | 0.125 | >64 | 0.5 | 2 |
| PRJEB42440 | AI2850 | *Klebsiella pneumoniae* | 151 | CAT02 | OXA-48 | ≤0.06 | ≤0.06 | ≤0.06 | ≤0.06 | ≤0.06 | ≤0.06 | 0.125 |
| PRJEB42440 | AI2851 | *Klebsiella pneumoniae* | 152 | CAT02 | OXA-48 | 32 | 0.125 | 0.25 | 0.125 | >64 | 4 | 1 |
| PRJEB42440 | AI2854 | *Klebsiella pneumoniae* | 152 | CAT02 | OXA-48 | 32 | 0.125 | 0.125 | ≤0.06 | >64 | 2 | 1 |
| PRJEB42440 | AN2364 | *Enterobacter cloacae complex* | 168 | GAL02 | OXA-48 | >64 | 0.5 | 2 | 1 | >64 | 1 | 1 |
| PRJEB42440 | AH0328 | *Enterobacter cloacae complex* | 171 | BAL01 | OXA-48 | 2 | 0.125 | 0.25 | 0.125 | 1 | 1 | 0.5 |
| PRJEB42440 | AI2844 | *Klebsiella pneumoniae* | 198 | CAT01 | OXA-48 | ≤0.06 | ≤0.06 | ≤0.06 | ≤0.06 | 0.25 | 0.125 | 0.5 |
| PRJEB42440 | AI2593 | *Klebsiella pneumoniae* | 219 | MAD01 | OXA-48 | 8 | 0.125 | 0.125 | 0.125 | 64 | 0.5 | 0.5 |
| PRJEB42440 | AI2699 | *Klebsiella oxytoca* | 325 | AST01 | OXA-48 | 2 | ≤0.06 | ≤0.06 | ≤0.06 | >64 | 0.25 | 0.5 |
| PRJEB42440 | AI2943 | *Klebsiella oxytoca* | 327 | AST01 | OXA-48 | ≤0.06 | ≤0.06 | ≤0.06 | ≤0.06 | 1 | 0.25 | 1 |
| PRJEB42440 | AI2637 | *Klebsiella pneumoniae* | 429 | CAT01 | OXA-48 | 16 | ≤0.06 | ≤0.06 | ≤0.06 | >64 | 1 | 0.5 |
| PRJEB42440 | AI2792 | *Klebsiella pneumoniae* | 437 | VAL01 | OXA-48 | 32 | 0.25 | 0.5 | 0.25 | >64 | 1 | 1 |
| PRJEB42440 | AI2667 | *Klebsiella pneumoniae* | 485 | AST01 | OXA-48 | ≤0.06 | ≤0.06 | 0.125 | ≤0.06 | 0.5 | 0.125 | 0.5 |
| PRJEB42440 | AN2346 | *Klebsiella pneumoniae* | 551 | GAL02 | OXA-48 | ≤0.06 | ≤0.06 | ≤0.06 | ≤0.06 | 0.5 | 0.125 | 2 |
| PRJEB42440 | AI2834 | *Escherichia coli* | 624 | VAL02 | OXA-48 | 2 | 0.5 | 1 | 1 | 4 | 1 | 1 |
| PRJEB42440 | AI2803 | *Klebsiella pneumoniae* | 628 | CAT04 | OXA-48 | 0.125 | 0.125 | 0.125 | 0.125 | 2 | 0.5 | 2 |
| PRJEB42440 | AI2904 | *Enterobacter cloacae complex* | 662 | MAD04 | OXA-48 | 16 | ≤0.06 | ≤0.06 | 0.125 | >64 | 2 | 0.25 |
| PRJEB42440 | AI2905 | *Enterobacter cloacae complex* | 732 | MAD04 | OXA-48 | 64 | 0.25 | 0.25 | 0.25 | 64 | 0.5 | 2 |
| PRJEB42440 | AI2672 | *Enterobacter cloacae complex* | 1379 | AST01 | OXA-48 | >64 | >64 | >64 | 64 | >64 | 2 | 4 |
| PRJEB42440 | AI2796 | *Enterobacter cloacae complex* | 1380 | CAT04 | OXA-48 | >64 | 16 | 8 | 4 | >64 | 1 | 1 |
| PRJEB42440 | AI2999 | *Enterobacter cloacae complex* | 1382 | CAT03 | OXA-48 | 2 | 0.125 | ≤0.06 | 0.125 | 32 | 0.5 | 0.5 |
| PRJEB42440 | AI2842 | *Klebsiella pneumoniae* | 1401 | CAT01 | OXA-48 | ≤0.06 | ≤0.06 | ≤0.06 | ≤0.06 | 0.5 | 0.5 | 0.25 |
| PRJEB53700 | 20220203 | *Escherichia coli* | 1722 | AND08 | OXA-244 | 1 | 0.125 | 0.125 | ≤0.06 | 1 | 0.125 | 0.5 |
| PRJEB53700 | 20220042 | *Klebsiella pneumoniae* | 1758 | AND02 | OXA-181 | ≤0.06 | ≤0.06 | ≤0.06 | ≤0.06 | >64 | 0.125 | 0.25 |
| PRJEB53700 | 20220160 | *Escherichia coli* | 2083 | AND11 | OXA-181 | >64 | >64 | >64 | >64 | >64 | 16 | 16 |
| PRJEB42440 | AI3027 | *Klebsiella pneumoniae* | 3362 | AND01 | OXA-48 | >64 | 0.25 | 0.5 | 0.5 | 32 | 1 | 1 |
| PRJEB42440 | AI3034 | *Klebsiella pneumoniae* | 4387 | AST01 | OXA-48 | 0.125 | ≤0.06 | ≤0.06 | ≤0.06 | 0.25 | 0.125 | 0.125 |
| PRJEB42440 | AI2740 | *Klebsiella pneumoniae* | 5000 | CAN01 | OXA-48 | 16 | ≤0.06 | ≤0.06 | ≤0.06 | >64 | 2 | 0.5 |
| PRJEB42440 | AI2907 | *Klebsiella pneumoniae* | 5001 | MAD04 | OXA-48 | 16 | ≤0.06 | 0.125 | 0.125 | >64 | 1 | 0.5 |
| PRJEB42440 | AI2974 | *Klebsiella pneumoniae* | 5002 | CLM01 | OXA-48 | 64 | 0.125 | 0.25 | ≤0.06 | 64 | 0.5 | 0.5 |
| PRJEB42440 | AI2994 | *Escherichia coli* | 11106 | CAT03 | OXA-48 | 0.25 | ≤0.06 | ≤0.06 | ≤0.06 | >64 | 0.5 | 0.25 |
| PRJEB53700 | 20200560 | *Escherichia coli* | 1049 | AND15 | OXA-48 | 0.25 | ≤0.06 | ≤0.06 | ≤0.06 | 1 | ≤0.06 | ≤0.06 |
| PRJEB53700 | 20211130 | *Klebsiella oxytoca* | 108 | AND07 | OXA-48 | 0.125 | ≤0.06 | ≤0.06 | ≤0.06 | 1 | 0.25 | 2 |
| PRJEB53700 | 20220523 | *Enterobacter cloacae* | 110 | AND17 | OXA-48 | 16 | 0.125 | ≤0.06 | 0.5 | 64 | 1 | 1 |
| PRJEB53700 | 20200300 | *Escherichia coli* | 11249 | AND02 | OXA-48 | 8 | ≤0.06 | 0.125 | 0.125 | 32 | 0.25 | 0.25 |
| PRJEB53700 | 20190736 | *Escherichia coli* | 1193 | AND07 | OXA-48 | 0.125 | ≤0.06 | 0.125 | ≤0.06 | 0.125 | ≤0.06 | 0.25 |
| PRJEB53700 | 20190666 | *Enterobacter cloacae* | 120 | AND07 | OXA-48 | 8 | 0.125 | 0.25 | 0.25 | 1 | 0.5 | 1 |
| PRJEB53700 | 20200049 | *Citrobacter freundii* | 125 | AND01 | OXA-48 | >64 | 1 | 1 | 1 | >64 | 0.5 | 1 |
| PRJEB53700 | 20190392 | *Klebsiella pneumoniae* | 1411 | AND07 | OXA-48 | 0.125 | ≤0.06 | ≤0.06 | 0.125 | 4 | 0.25 | 1 |
| PRJEB53700 | 20190733 | *Klebsiella pneumoniae* | 1563 | AND07 | OXA-48 | ≤0.06 | ≤0.06 | ≤0.06 | ≤0.06 | 0.5 | 0.25 | 1 |
| PRJEB53700 | 20200182 | *Klebsiella pneumoniae* | 1565 | AND07 | OXA-48 | ≤0.06 | ≤0.06 | ≤0.06 | ≤0.06 | 0.25 | ≤0.06 | 0.25 |
| PRJEB53700 | 20200206 | *Escherichia coli* | 1582 | AND07 | OXA-48 | 0.125 | ≤0.06 | ≤0.06 | ≤0.06 | 0.125 | ≤0.06 | 0.25 |
| PRJEB53700 | 20210142 | *Enterobacter cloacae* | 1599 | AND07 | OXA-48 | 32 | 0.25 | 0.25 | 0.125 | >64 | 1 | 2 |
| PRJEB53700 | 20200179 | *Klebsiella pneumoniae* | 17 | AND07 | OXA-48 | 0.125 | ≤0.06 | ≤0.06 | ≤0.06 | 0.25 | 0.125 | 0.25 |
| PRJEB53700 | 20210183 | *Escherichia coli* | 58 | AND11 | OXA-48 | ≤0.06 | ≤0.06 | ≤0.06 | ≤0.06 | ≤0.06 | ≤0.06 | 0.125 |
| PRJEB53700 | 20220475 | *Klebsiella pneumoniae* | 23 | AND18 | OXA-48 | >64 | 0.25 | 1 | 0.5 | >64 | 2 | 8 |
| CTB: ceftibuten; CTB/A: ceftibuten/avibactam; CTB/L: ceftibuten/ledaborbactam; CTB/X: ceftibuten/xeruborbactam; CAZ: ceftazidime; CAZ/A: ceftazidime/avibactam; MEM: meropenem.  ^a^ EUCAST breakpoints indicated for Enterobacterales | | | | | | | | | | | | |

| **Table S4.** Antimicrobial susceptibility data for ceftibuten combinations and comparator agents against KPC-producing Enterobacterales (n=100). | | | | | | | | | | | | |
| --- | --- | --- | --- | --- | --- | --- | --- | --- | --- | --- | --- | --- |
| **BioProject ID** | **Genome number** | **Species** | **MLST** | **Hospital code** | **Carbapenemase** | **MIC (mg/L)^a^** | | | | | | |
|  |  |  |  |  |  | **CTB**  **(R>1)** | **CTB/A**  **(R>1)** | **CTB/L**  **(R>1)** | **CTB/X**  **(R>1)** | **CAZ**  **(R>4)** | **CAZ/A**  **(R>8)** | **MEM**  **(R>8)** |
| PRJEB42440 | AI2588 | *Klebsiella pneumoniae* | 512 | MAD01 | KPC-3 | 8 | ≤0.06 | 0.125 | ≤0.06 | >64 | 4 | 16 |
| PRJEB42440 | AI2602 | *Citrobacter freundii* | 112 | MAD01 | KPC-2 | 32 | 0.125 | 0.125 | 0.125 | >64 | 1 | 16 |
| PRJEB42440 | AI2826 | *Citrobacter freundii* | 22 | MAD01 | KPC-2 | 64 | 0.5 | 0.125 | 0.5 | >64 | 1 | 8 |
| PRJEB42440 | AI2614 | *Klebsiella oxytoca* | 324 | CYL01 | KPC-2 | 64 | ≤0.06 | ≤0.06 | ≤0.06 | >64 | 2 | 2 |
| PRJEB42440 | AI2835 | *Klebsiella pneumoniae* | 512 | AND03 | KPC-3 | 32 | 0.25 | 0.5 | 0.25 | >64 | 4 | 64 |
| PRJEB42440 | AI2627 | *Klebsiella pneumoniae* | 512 | AND03 | KPC-3 | 16 | 0.25 | 0.125 | 0.125 | >64 | 16 | >64 |
| PRJEB42440 | AI2628 | *Klebsiella pneumoniae* | 512 | AND03 | KPC-3 | 16 | 0.25 | 0.25 | 0.125 | >64 | 8 | >64 |
| PRJEB42440 | AI2629 | *Klebsiella pneumoniae* | 512 | AND03 | KPC-3 | 16 | 0.125 | ≤0.06 | ≤0.06 | >64 | 4 | 32 |
| PRJEB42440 | AI2631 | *Klebsiella pneumoniae* | 512 | AND03 | KPC-3 | 32 | 0.25 | 0.25 | 0.25 | >64 | 16 | >64 |
| PRJEB42440 | AI2836 | *Klebsiella pneumoniae* | 512 | AND03 | KPC-3 | 16 | 0.125 | 0.25 | 0.25 | >64 | 8 | >64 |
| PRJEB42440 | AI2632 | *Klebsiella pneumoniae* | 512 | AND03 | KPC-3 | 16 | 0.25 | 0.25 | 0.25 | >64 | 8 | >64 |
| PRJEB42440 | AI2837 | *Klebsiella pneumoniae* | 512 | AND03 | KPC-3 | 16 | 0.25 | 0.25 | 0.25 | >64 | 8 | >64 |
| PRJEB42440 | AI2643 | *Klebsiella pneumoniae* | 307 | MAD03 | KPC-3 | 2 | ≤0.06 | ≤0.06 | ≤0.06 | >64 | 0.5 | 2 |
| PRJEB42440 | AI2644 | *Klebsiella pneumoniae* | 307 | MAD03 | KPC-3 | 8 | ≤0.06 | 0.125 | ≤0.06 | >64 | 2 | 2 |
| PRJEB42440 | AI2645 | *Klebsiella pneumoniae* | 307 | MAD03 | KPC-3 | 8 | ≤0.06 | ≤0.06 | ≤0.06 | >64 | 2 | 2 |
| PRJEB42440 | AI2883 | *Klebsiella pneumoniae* | 307 | MAD03 | KPC-3 | 4 | ≤0.06 | ≤0.06 | ≤0.06 | >64 | 0.5 | 4 |
| PRJEB42440 | AI2889 | *Klebsiella pneumoniae* | 307 | MAD03 | KPC-3 | 8 | ≤0.06 | ≤0.06 | ≤0.06 | >64 | 2 | 4 |
| PRJEB42440 | AI2646 | *Klebsiella pneumoniae* | 307 | MAD03 | KPC-3 | 16 | ≤0.06 | 0.125 | ≤0.06 | >64 | 1 | 4 |
| PRJEB42440 | AI2647 | *Klebsiella pneumoniae* | 307 | MAD03 | KPC-3 | 8 | ≤0.06 | ≤0.06 | ≤0.06 | >64 | 2 | 2 |
| PRJEB42440 | AI2648 | *Klebsiella pneumoniae* | 307 | MAD03 | KPC-3 | 16 | ≤0.06 | ≤0.06 | ≤0.06 | >64 | 2 | 2 |
| PRJEB42440 | AI2649 | *Klebsiella pneumoniae* | 307 | MAD03 | KPC-3 | 16 | ≤0.06 | 0.125 | 0.125 | >64 | 1 | 2 |
| PRJEB42440 | AI2650 | *Klebsiella pneumoniae* | 307 | MAD03 | KPC-3 | 8 | ≤0.06 | ≤0.06 | ≤0.06 | >64 | 1 | ≤0.06 |
| PRJEB42440 | AI2651 | *Klebsiella pneumoniae* | 459 | MAD03 | KPC-3 | 8 | ≤0.06 | 0.125 | ≤0.06 | >64 | 2 | 8 |
| PRJEB42440 | AI2652 | *Klebsiella pneumoniae* | 307 | MAD03 | KPC-3 | 16 | ≤0.06 | ≤0.06 | ≤0.06 | >64 | 4 | 2 |
| PRJEB42440 | AI2653 | *Klebsiella pneumoniae* | 307 | MAD03 | KPC-3 | 8 | ≤0.06 | ≤0.06 | ≤0.06 | 16 | 1 | 4 |
| PRJEB42440 | AI2901 | *Klebsiella pneumoniae* | 307 | MAD03 | KPC-3 | 2 | ≤0.06 | ≤0.06 | ≤0.06 | >64 | 2 | 8 |
| PRJEB42440 | AI2654 | *Klebsiella pneumoniae* | 307 | MAD03 | KPC-3 | 8 | ≤0.06 | ≤0.06 | ≤0.06 | 64 | 1 | 2 |
| PRJEB42440 | AI2655 | *Klebsiella pneumoniae* | 307 | MAD03 | KPC-3 | 8 | ≤0.06 | 0.125 | ≤0.06 | >64 | 1 | 2 |
| PRJEB42440 | AI2940 | *Klebsiella pneumoniae* | 258 | BAL01 | KPC-3 | 32 | 0.25 | 0.5 | 0.25 | >64 | 4 | 32 |
| PRJEB42440 | AI2805 | *Klebsiella pneumoniae* | 512 | AND04 | KPC-3 | 16 | 0.25 | 0.25 | 0.25 | >64 | 32 | >64 |
| PRJEB42440 | AI2806 | *Klebsiella pneumoniae* | 512 | AND04 | KPC-3 | 16 | 0.25 | 0.25 | 0.5 | >64 | 8 | 64 |
| PRJEB42440 | AI2807 | *Klebsiella pneumoniae* | 512 | AND04 | KPC-3 | 32 | 0.125 | 0.25 | 0.25 | >64 | 8 | >64 |
| PRJEB42440 | AI2808 | *Klebsiella pneumoniae* | 512 | AND04 | KPC-3 | 32 | 0.5 | 0.25 | 0.25 | >64 | 8 | 64 |
| PRJEB42440 | AI2809 | *Klebsiella pneumoniae* | 512 | AND04 | KPC-3 | 8 | 0.125 | 0.125 | 0.125 | >64 | 4 | 64 |
| PRJEB42440 | AI2810 | *Klebsiella pneumoniae* | 512 | AND04 | KPC-3 | 32 | 0.25 | 0.25 | 0.25 | 16 | 16 | >64 |
| PRJEB42440 | AI2811 | *Klebsiella pneumoniae* | 512 | AND04 | KPC-3 | 32 | 0.25 | 0.25 | 0.25 | >64 | 1 | >64 |
| PRJEB42440 | AI2812 | *Klebsiella pneumoniae* | 512 | AND04 | KPC-3 | 32 | 0.25 | 0.5 | 0.25 | >64 | 8 | >64 |
| PRJEB42440 | AI2813 | *Klebsiella pneumoniae* | 512 | AND04 | KPC-3 | 16 | ≤0.06 | 0.125 | 0.125 | >64 | 32 | 64 |
| PRJEB42440 | AI2814 | *Klebsiella pneumoniae* | 512 | AND04 | KPC-3 | 32 | 0.25 | 0.5 | 0.125 | >64 | 8 | >64 |
| PRJEB42440 | AI2815 | *Klebsiella pneumoniae* | 512 | AND04 | KPC-3 | 32 | 0.125 | 0.25 | 0.25 | >64 | 8 | >64 |
| PRJEB42440 | AI2816 | *Klebsiella pneumoniae* | 512 | AND04 | KPC-3 | 16 | 0.125 | 0.25 | 0.25 | >64 | 8 | >64 |
| PRJEB42440 | AI2817 | *Klebsiella pneumoniae* | 512 | AND04 | KPC-3 | 64 | 0.25 | 0.5 | 0.25 | >64 | 8 | >64 |
| PRJEB53700 | 20220366 | *Klebsiella pneumoniae* | 512 | AND01 | KPC-132 | 32 | 1 | 1 | 0.5 | >64 | >64 | 16 |
| PRJEB53700 | 20220166 | *Klebsiella pneumoniae* | 512 | AND14 | KPC-23 | 8 | ≤0.06 | ≤0.06 | ≤0.06 | >64 | 16 | 4 |
| PRJEB53700 | 20220294 | *Klebsiella pneumoniae* | 512 | AND14 | KPC-66 | 16 | 0.5 | 2 | 2 | >64 | 32 | 4 |
| PRJEB53700 | 20211109 | *Klebsiella pneumoniae* | 512 | AND03 | KPC-31 | 16 | 1 | 0.5 | 0.125 | >64 | >64 | 2 |
| PRJEB53700 | 20220595 | *Klebsiella oxytoca* | 108 | AND18 | KPC-2 | 4 | ≤0.06 | ≤0.06 | ≤0.06 | >64 | 1 | 2 |
| PRJEB53700 | 20190510 | *Klebsiella pneumoniae* | 307 | AND09 | KPC-2 | 64 | ≤0.06 | 0.125 | 0.125 | >64 | 2 | 0.5 |
| PRJEB53700 | 20190576 | *Citrobacter freundii* | 8 | AND06 | KPC-2 | >64 | 0.25 | 0.25 | 0.5 | >64 | 0.5 | 2 |
| PRJEB53700 | 20190661 | *Enterobacter cloacae* | 350 | AND03 | KPC-2 | 4 | ≤0.06 | 0.125 | ≤0.06 | 8 | 0.25 | 1 |
| PRJEB53700 | 20190771 | *Klebsiella pneumoniae* | 4973 | AND08 | KPC-2 | >64 | 0.25 | 0.5 | 0.25 | 64 | 0.5 | 2 |
| PRJEB53700 | 20200684 | *Citrobacter freundii* | 18 | AND17 | KPC-3 | >64 | 0.25 | ≤0.06 | 0.25 | >64 | 4 | 4 |
| PRJEB53700 | 20200614 | *Klebsiella pneumoniae* | 307 | AND01 | KPC-3 | 32 | 0.125 | 0.25 | ≤0.06 | >64 | 2 | 16 |
| PRJEB53700 | 20200474 | *Klebsiella pneumoniae* | 15 | AND17 | KPC-2 | 4 | ≤0.06 | ≤0.06 | ≤0.06 | 64 | 2 | 8 |
| PRJEB53700 | 20200479 | *Escherichia coli* | 357 | AND02 | KPC-2 | 8 | ≤0.06 | ≤0.06 | ≤0.06 | 64 | 0.25 | 2 |
| PRJEB53700 | 20220707 | *Klebsiella pneumoniae* | 258 | AND07 | KPC-3 | 32 | 0.25 | 0.5 | 0.25 | >64 | 2 | 16 |
| PRJEB53700 | 20220496 | *Klebsiella pneumoniae* | 35 | AND14 | KPC-3 | 4 | ≤0.06 | 0.125 | ≤0.06 | >64 | 2 | 8 |
| PRJEB53700 | 20220247 | *Citrobacter freundii* | 730 | AND06 | KPC-2 | 64 | 0.125 | 1 | 0.125 | >64 | 0.5 | 1 |
| PRJEB53700 | 20220292 | *Klebsiella pneumoniae* | 512 | AND14 | KPC-3 | >64 | 1 | 0.5 | 0.125 | >64 | 16 | >64 |
| PRJEB53700 | 20210037 | *Escherichia coli* | 648 | AND17 | KPC-2 | 32 | ≤0.06 | ≤0.06 | ≤0.06 | 32 | 0.125 | 2 |
| PRJEB53700 | 20210342 | *Klebsiella oxytoca* | 170 | AND12 | KPC-2 | 0.5 | ≤0.06 | ≤0.06 | ≤0.06 | 16 | 0.5 | 1 |
| PRJEB53700 | 20200356 | *Klebsiella pneumoniae* | 258 | AND16 | KPC-2 | 16 | ≤0.06 | 0.125 | ≤0.06 | >64 | 2 | 32 |
| PRJEB53700 | 20210847 | *Escherichia coli* | 327 | AND18 | KPC-2 | 16 | 0.25 | ≤0.06 | ≤0.06 | >64 | 1 | 1 |
| PRJEB53700 | 20210607 | *Enterobacter cloacae* | 93 | AND13 | KPC-2 | >64 | 4 | 2 | 1 | >64 | 2 | 16 |
| PRJNA1133624 | ARGA00246 | *Klebsiella pneumoniae* | 307 | GAL02 | KPC-2 | 16 | ≤0.06 | ≤0.06 | ≤0.06 | >64 | 1 | 4 |
| PRJNA1133624 | ARGA00247 | *Klebsiella pneumoniae* | 512 | GAL02 | KPC-3 | 8 | 0.125 | 0.125 | 0.125 | >64 | 4 | 16 |
| PRJNA1133624 | ARGA00248 | *Escherichia coli* | 506 | GAL02 | KPC-2 | 0.5 | ≤0.06 | ≤0.06 | ≤0.06 | 2 | 0.125 | 0.5 |
| PRJNA1133624 | ARGA00260 | *Klebsiella pneumoniae* | 307 | GAL02 | KPC-2 | 32 | ≤0.06 | ≤0.06 | ≤0.06 | >64 | 2 | 8 |
| PRJNA1133624 | ARGA00262 | *Citrobacter freundii* | 155 | GAL02 | KPC-2 | >64 | 32 | 64 | >64 | >64 | 4 | >64 |
| PRJNA1133624 | ARGA00269 | *Klebsiella pneumoniae* | 512 | GAL02 | KPC-3 | 8 | 0.125 | 0.125 | ≤0.06 | >64 | 2 | 16 |
| PRJNA1133624 | ARGA00346 | *Klebsiella pneumoniae* | 512 | GAL02 | KPC-3 | 16 | 0.25 | 0.25 | 0.25 | 8 | 1 | 0.25 |
| PRJEB42440 | AI2700 | *Klebsiella pneumoniae* | 1961 | GAL02 | KPC-2 | 2 | ≤0.06 | 0.125 | ≤0.06 | 32 | 1 | 2 |
| PRJEB42440 | AI2701 | *Klebsiella pneumoniae* | 273 | GAL02 | KPC-2 | 8 | 0.25 | 0.25 | 0.25 | 32 | 1 | 64 |
| PRJEB42440 | AN2340 | *Klebsiella pneumoniae* | 2295 | GAL02 | KPC-2 | 0.25 | ≤0.06 | ≤0.06 | ≤0.06 | 4 | 0.125 | 1 |
| PRJEB42440 | AI2703 | *Escherichia coli* | 162 | GAL02 | KPC-2 | 8 | ≤0.06 | ≤0.06 | ≤0.06 | 32 | 0.5 | 8 |
| PRJEB42440 | AI2704 | *Enterobacter cloacae* | 96 | GAL02 | KPC-2 | 32 | 0.125 | 0.25 | 0.125 | >64 | 2 | 32 |
| PRJEB42440 | AI2979 | *Klebsiella pneumoniae* | 1961 | GAL02 | KPC-2 | 2 | ≤0.06 | ≤0.06 | ≤0.06 | 32 | 0.5 | 2 |
| PRJEB42440 | AI2705 | *Enterobacter cloacae* | 515 | GAL02 | KPC-2 | >64 | 1 | 0.25 | 2 | 64 | 0.5 | 16 |
| PRJEB42440 | AI2706 | *Klebsiella pneumoniae* | 512 | GAL02 | KPC-3 | 16 | 0.125 | 0.25 | 0.25 | >64 | 8 | >64 |
| PRJEB42440 | AI2707 | *Klebsiella pneumoniae* | 512 | GAL02 | KPC-3 | 16 | ≤0.06 | 0.25 | 0.125 | >64 | 4 | 64 |
| PRJNA1133624 | ARGA00426 | *Citrobacter freundii* | 85 | GAL02 | KPC-2 | 8 | 0.125 | 0.125 | 0.125 | 32 | 0.5 | 64 |
| PRJEB42440 | AI2709 | *Klebsiella pneumoniae* | 678 | GAL02 | KPC-3 | 4 | ≤0.06 | ≤0.06 | ≤0.06 | >64 | 8 | 8 |
| PRJEB42440 | AI2710 | *Enterobacter cloacae* | 96 | GAL02 | KPC-2 | 32 | 0.25 | 0.5 | 0.125 | >64 | 2 | 64 |
| PRJEB42440 | AI2711 | *Klebsiella oxytoca* | N/A | GAL02 | KPC-2 | 2 | ≤0.06 | ≤0.06 | ≤0.06 | 8 | 1 | 8 |
| PRJEB42440 | AI2980 | *Klebsiella pneumoniae* | 1961 | GAL02 | KPC-2 | 2 | ≤0.06 | ≤0.06 | ≤0.06 | 16 | 1 | 2 |
| PRJEB42440 | AI2981 | *Escherichia coli* | 10 | GAL02 | KPC-2 | 1 | ≤0.06 | ≤0.06 | ≤0.06 | 8 | 0.125 | 0.125 |
| PRJEB42440 | AI2982 | *Klebsiella pneumoniae* | 1961 | GAL02 | KPC-2 | 2 | ≤0.06 | ≤0.06 | ≤0.06 | 64 | 0.5 | 4 |
| PRJEB42440 | AI2713 | *Klebsiella pneumoniae* | 273 | GAL02 | KPC-2 | 16 | ≤0.06 | 0.125 | ≤0.06 | 64 | 0.5 | >64 |
| PRJEB42440 | AI2714 | *Klebsiella pneumoniae* | 512 | GAL02 | KPC-3 | 8 | 0.125 | 0.25 | 0.125 | >64 | 2 | 64 |
| PRJEB42440 | AI2715 | *Escherichia coli* | 23 | GAL02 | KPC-2 | 4 | ≤0.06 | 0.125 | ≤0.06 | 64 | 0.25 | 8 |
| PRJEB42440 | AI2983 | *Enterobacter cloacae* | 1381 | GAL02 | KPC-2 | 16 | 8 | 0.125 | 0.25 | 64 | 16 | 16 |
| PRJEB42440 | AI2716 | *Escherichia coli* | 131 | GAL02 | KPC-3 | 8 | 0.5 | ≤0.06 | ≤0.06 | 0.25 | 0.125 | ≤0.06 |
| PRJEB42440 | AI2717 | *Klebsiella pneumoniae* | 1961 | GAL02 | KPC-2 | 8 | 1 | ≤0.06 | ≤0.06 | 32 | 0.5 | 4 |
| PRJEB42440 | AI2984 | *Enterobacter cloacae* | 515 | GAL02 | KPC-2 | >64 | 2 | 0.5 | 2 | 16 | 0.5 | >64 |
| PRJEB42440 | AI2719 | *Enterobacter cloacae* | 515 | GAL02 | KPC-2 | >64 | 1 | 0.25 | 2 | 8 | 0.5 | 0.25 |
| PRJEB42440 | AI2985 | *Klebsiella pneumoniae* | 1961 | GAL02 | KPC-2 | 4 | ≤0.06 | 0.125 | ≤0.06 | 16 | 1 | 4 |
| PRJEB42440 | AI3060 | *Klebsiella pneumoniae* | 1961 | GAL02 | KPC-2 | 8 | 0.125 | 0.125 | 0.125 | 16 | 1 | 4 |
| PRJEB42440 | AI2721 | *Escherichia coli* | 131 | GAL02 | KPC-2 | 1 | ≤0.06 | ≤0.06 | ≤0.06 | 4 | ≤0.06 | 0.25 |
| PRJEB42440 | AI2722 | *Klebsiella pneumoniae* | 307 | GAL02 | KPC-3 | 4 | ≤0.06 | ≤0.06 | ≤0.06 | >64 | 1 | 4 |
| PRJEB42440 | AI2723 | *Klebsiella pneumoniae* | 258 | GAL02 | KPC-3 | 4 | ≤0.06 | ≤0.06 | ≤0.06 | >64 | 2 | 16 |
| CTB: ceftibuten; CTB/A: ceftibuten/avibactam; CTB/L: ceftibuten/ledaborbactam; CTB/X: ceftibuten/xeruborbactam; CAZ: ceftazidime; CAZ/A: ceftazidime/avibactam; MEM: meropenem.  ^a^ EUCAST breakpoints indicated for Enterobacterales | | | | | | | | | | | | |

| **Table S5.** Antimicrobial susceptibility data for ceftibuten combinations and comparator agents against MBL-producing Enterobacterales (n=100). | | | | | | | | | | | | |
| --- | --- | --- | --- | --- | --- | --- | --- | --- | --- | --- | --- | --- |
| **BioProject ID** | **Genome number** | **Species** | **MLST** | **Hospital code** | **Carbapenemase** | **MIC (mg/L)^a^** | | | | | | |
|  |  |  |  |  |  | **CTB**  **(R>1)** | **CTB/A**  **(R>1)** | **CTB/L**  **(R>1)** | **CTB/X**  **(R>1)** | **CAZ**  **(R>4)** | **CAZ/A**  **(R>8)** | **MEM**  **(R>8)** |
| PRJEB42440 | AI2991 | *Klebsiella oxytoca* | 36 | NAV01 | VIM-1 | 8 | 4 | 1 | ≤0.06 | >64 | 64 | 0.125 |
| PRJEB42440 | AI2992 | *Enterobacter cloacae complex* | 96 | NAV01 | IMP-8 | >64 | 64 | 64 | 8 | >64 | >64 | 0.25 |
| PRJEB42440 | AI2839 | *Enterobacter cloacae complex* | 413 | CAT01 | VIM-1 | 64 | 64 | 64 | 2 | >64 | >64 | 1 |
| PRJEB42440 | AI2843 | *Escherichia coli* | 410 | CAT01 | NDM-5 | >64 | >64 | >64 | >64 | >64 | >64 | 4 |
| PRJEB42440 | AI2848 | *Klebsiella pneumoniae* | 1083 | CAT01 | VIM-1 | 32 | 32 | 4 | ≤0.06 | >64 | >64 | 0.25 |
| PRJEB42440 | AI2972 | *Enterobacter cloacae complex* | 114 | CLM01 | VIM-1 | >64 | 32 | 8 | 0.5 | >64 | >64 | 0.25 |
| PRJEB42440 | AI2626 | *Klebsiella pneumoniae* | 11 | AND03 | VIM-1 | 64 | 32 | 16 | 1 | >64 | 64 | 0.5 |
| PRJEB42440 | AI2772 | *Enterobacter cloacae complex* | 110 | CAT03 | VIM-1 | >64 | 64 | 16 | 1 | >64 | >64 | 0.5 |
| PRJEB42440 | AI2859 | *Enterobacter cloacae complex* | 133 | MAD02 | VIM-1 | 64 | 32 | 16 | 0.25 | >64 | >64 | 1 |
| PRJEB42440 | AI2860 | *Escherichia coli* | 602 | MAD02 | VIM-1 | 8 | 4 | 4 | ≤0.06 | >64 | 64 | ≤0.06 |
| PRJEB39112 | AI2830 | *Klebsiella pneumoniae* | 147 | GAL03 | NDM-1 | >64 | >64 | >64 | 8 | >64 | >64 | 4 |
| PRJEB42440 | AI2828 | *Klebsiella oxytoca* | 202 | ARA01 | VIM-1 | 64 | 64 | 8 | 0.25 | >64 | >64 | 0.25 |
| PRJEB42440 | AI2858 | *Escherichia coli* | 648 | CAT02 | NDM-7 | >64 | >64 | >64 | >64 | >64 | >64 | 32 |
| PRJEB42440 | AI2880 | *Klebsiella pneumoniae* | 101 | MAD03 | NDM-1 | >64 | >64 | >64 | 8 | >64 | >64 | 8 |
| PRJEB42440 | AI2885 | *Klebsiella pneumoniae* | 307 | MAD03 | VIM-1 | 32 | 16 | 16 | 0.125 | >64 | >64 | 0.25 |
| PRJEB39112 | AI2898 | *Klebsiella pneumoniae* | 101 | MAD03 | NDM-1 | >64 | >64 | >64 | 4 | >64 | >64 | 4 |
| PRJEB42440 | AI2660 | *Klebsiella pneumoniae* | 11 | BAL01 | VIM-1 | 64 | 32 | 32 | 0.25 | >64 | >64 | 0.5 |
| PRJEB42440 | AI2779 | *Klebsiella pneumoniae* | 437 | VAL01 | NDM-23 | >64 | >64 | >64 | 4 | >64 | >64 | 2 |
| PRJEB39112 | AI3007 | *Klebsiella pneumoniae* | 101 | VAL01 | NDM-1 | >64 | >64 | >64 | 8 | >64 | >64 | 4 |
| PRJEB42440 | AI2793 | *Klebsiella pneumoniae* | 437 | VAL01 | NDM-1 | >64 | >64 | >64 | 2 | >64 | >64 | 4 |
| PRJEB42440 | AI2797 | *Enterobacter cloacae complex* | 24 | CAT04 | VIM-1 | 64 | 64 | 32 | 0.5 | >64 | >64 | 1 |
| PRJEB42440 | AI2798 | *Enterobacter cloacae complex* | 78 | CAT04 | VIM-1 | >64 | >64 | 64 | 4 | >64 | >64 | 1 |
| PRJEB42440 | AI3012 | *Klebsiella oxytoca* | 2 | CAT04 | VIM-1 | 32 | 32 | 32 | 0.125 | >64 | >64 | 0.5 |
| PRJEB42440 | AI3013 | *Enterobacter cloacae complex* | 764 | CAT04 | VIM-1 | 64 | 64 | 32 | 2 | >64 | >64 | 1 |
| PRJEB39112 | AI2934 | *Klebsiella pneumoniae* | 395 | AND02 | NDM-1 | >64 | >64 | >64 | 16 | >64 | >64 | 8 |
| PRJEB53686 | 20190525 | *Klebsiella pneumoniae* | 147 | AND10 | NDM-1 | >64 | >64 | >64 | 8 | >64 | >64 | 8 |
| PRJEB53700 | 20190050 | *Klebsiella pneumoniae* | 11 | AND06 | NDM-7 | >64 | >64 | >64 | 16 | >64 | >64 | 32 |
| PRJEB53686 | 20190123 | *Klebsiella pneumoniae* | 1873 | AND07 | IMP-8 | 32 | 32 | 32 | 2 | >64 | >64 | 0.5 |
| PRJEB53686 | 20190430 | *Escherichia coli* | 10087 | AND12 | NDM-7 | >64 | >64 | >64 | 4 | >64 | >64 | 8 |
| PRJEB53700 | 20190018 | *Klebsiella oxytoca* | 2 | AND05 | IMP-8 | >64 | >64 | >64 | 8 | >64 | >64 | 0.5 |
| PRJEB53686 | 20190067 | *Klebsiella pneumoniae* | 464 | AND08 | IMP-8 | >64 | >64 | >64 | 16 | >64 | >64 | 1 |
| PRJEB53700 | 20190130 | *Escherichia coli* | 216 | AND11 | VIM-1 | 16 | 8 | 4 | ≤0.06 | 16 | 8 | 0.5 |
| PRJEB53686 | 20190856 | *Klebsiella pneumoniae* | 307 | AND16 | NDM-7 | >64 | >64 | >64 | 8 | >64 | >64 | 16 |
| PRJEB53700 | 20200592 | *Klebsiella pneumoniae* | 234 | AND08 | VIM-1 | >64 | 64 | 64 | 0.5 | >64 | >64 | 2 |
| PRJEB53700 | 20211129 | *Enterobacter cloacae* | 1381 | AND17 | VIM-4 | >64 | 64 | 16 | 1 | 16 | 16 | 1 |
| PRJEB53700 | 20210949 | *Klebsiella pneumoniae* | 219 | AND08 | VIM-1 | >64 | 64 | 32 | 0.5 | >64 | >64 | 2 |
| PRJEB53700 | 20220763 | *Escherichia coli* | 12279 | AND18 | NDM-1 | >64 | >64 | 64 | 2 | >64 | >64 | 1 |
| PRJEB53700 | 20220823 | *Klebsiella pneumoniae* | 15 | AND07 | NDM-5 | >64 | >64 | >64 | 2 | >64 | >64 | 4 |
| PRJEB53700 | 20220579 | *Escherichia coli* | 1193 | AND08 | VIM-1 | >64 | >64 | 64 | 2 | >64 | >64 | 0.25 |
| PRJEB53700 | 20220548 | *Escherichia coli* | 405 | AND11 | NDM-1 | >64 | >64 | >64 | >64 | >64 | >64 | 16 |
| PRJEB53700 | 20220085 | *Escherichia coli* | 167 | AND15 | NDM-5 | >64 | >64 | >64 | 64 | >64 | >64 | 2 |
| PRJEB53700 | 20220343 | *Escherichia coli* | 69 | AND08 | VIM-1 | >64 | 64 | 64 | 2 | >64 | >64 | 0.5 |
| PRJEB53700 | 20210070 | *Klebsiella pneumoniae* | 1805 | AND18 | VIM-33 | 64 | 64 | 64 | 2 | >64 | >64 | >64 |
| PRJEB53700 | 20210320 | *Escherichia coli* | 744 | AND18 | VIM-1 | 64 | 64 | 64 | 1 | >64 | >64 | 0.5 |
| PRJEB53700 | 20210746 | *Klebsiella pneumoniae* | 15 | AND17 | NDM-5 | >64 | >64 | 64 | 4 | >64 | >64 | 8 |
| PRJEB53700 | 20200150 | *Escherichia coli* | 10 | AND02 | VIM-1 | 4 | 4 | 2 | ≤0.06 | 64 | 32 | ≤0.06 |
| PRJEB53700 | 20200044 | *Enterobacter cloacae* | 1397 | AND01 | NDM-1 | >64 | 64 | 64 | 2 | >64 | >64 | 1 |
| PRJEB53700 | 20210672 | *Enterobacter cloacae* | 96 | AND03 | IMP-22 | 64 | 64 | 32 | 4 | >64 | >64 | 2 |
| PRJEB53700 | 20210743 | *Escherichia coli* | 162 | AND06 | NDM-1 | >64 | >64 | 32 | 2 | >64 | >64 | 1 |
| PRJNA1216752 | I399 | *Klebsiella pneumoniae* | 147 | ICA01 | NDM-14 | >64 | >64 | >64 | 64 | >64 | >64 | >64 |
| PRJNA1133624 | ARGA00135 | *Raoultella planticola* | N/A | GAL02 | VIM-1 | 64 | 32 | 32 | 0.5 | 64 | 64 | 32 |
| PRJNA1133624 | ARGA00197 | *Escherichia coli* | 10 | GAL02 | VIM-1 | 16 | 16 | 8 | ≤0.06 | >64 | >64 | 0.125 |
| PRJNA1133624 | ARGA00198 | *Enterobacter cloacae* | 3297 | GAL02 | VIM-1 | >64 | 32 | 16 | 1 | >64 | >64 | 0.25 |
| PRJNA1133624 | ARGA00245 | *Enterobacter hormaechei* | 102 | GAL02 | VIM-1 | >64 | 64 | 64 | 8 | >64 | >64 | 4 |
| PRJNA1133624 | ARGA00259 | *Enterobacter hormaechei* | 102 | GAL02 | VIM-1 | >64 | 64 | 64 | 4 | >64 | >64 | 8 |
| PRJNA1133624 | ARGA00270 | *Enterobacter hormaechei* | 102 | GAL02 | VIM-1 | >64 | 64 | 64 | 4 | >64 | >64 | 1 |
| PRJNA1133624 | ARGA00078 | *Klebsiella pneumoniae* | 147 | GAL02 | NDM-1 | >64 | 64 | 64 | 16 | >64 | >64 | 64 |
| PRJNA1133624 | ARGA00152 | *Klebsiella pneumoniae* | 6849 | GAL02 | NDM-1 | >64 | >64 | >64 | 4 | >64 | >64 | 4 |
| PRJNA1133624 | ARGA00195 | *Klebsiella aerogenes* | 93 | GAL02 | NDM-1 | >64 | >64 | 64 | 2 | >64 | >64 | 8 |
| PRJNA1133624 | ARGA00205 | *Klebsiella pneumoniae* | 17 | GAL02 | NDM-1 | 64 | 64 | 64 | 2 | >64 | >64 | 4 |
| PRJNA1133624 | ARGA00265 | *Klebsiella pneumoniae* | 147 | GAL02 | NDM-1 | >64 | >64 | 64 | 2 | >64 | >64 | 4 |
| PRJNA1133624 | ARGA00318 | *Klebsiella pneumoniae* | 11 | GAL02 | NDM-5 | >64 | >64 | >64 | 16 | >64 | >64 | 16 |
| PRJNA1133624 | ARGA00373 | *Proteus rettgeri* | 4 | GAL02 | NDM-1 | 4 | 4 | 2 | 0.25 | >64 | >64 | 4 |
| PRJNA1133624 | ARGA00391 | *Enterobacter cloacae* | 1718 | GAL02 | NDM-5 | >64 | >64 | >64 | 8 | >64 | >64 | 32 |
| PRJEB53700 | 20220856 | *Klebsiella pneumoniae* | 11 | AND11 | VIM-1 | >64 | >64 | 32 | 2 | >64 | >64 | 0.5 |
| PRJEB53700 | 20220459 | *Klebsiella pneumoniae* | 15 | AND02 | VIM-1 | 64 | 64 | 16 | 0.25 | >64 | >64 | 0.5 |
| PRJEB53700 | 20210875 | *Klebsiella pneumoniae* | 20 | AND08 | VIM-1 | >64 | 32 | 16 | 0.25 | >64 | >64 | 0.5 |
| PRJEB53700 | 20220344 | *Citrobacter freundii* | 22 | AND17 | VIM-1 | >64 | 16 | 16 | 0.5 | >64 | >64 | 0.25 |
| PRJEB53700 | 20190530 | *Klebsiella pneumoniae* | 25 | AND08 | VIM-1 | >64 | 64 | 16 | 0.5 | >64 | >64 | 0.5 |
| PRJEB53700 | 20210679 | *Klebsiella pneumoniae* | 35 | AND08 | VIM-1 | >64 | 64 | 32 | 2 | >64 | >64 | 8 |
| PRJEB53700 | 20210479 | *Klebsiella pneumoniae* | 37 | AND08 | VIM-1 | 32 | 32 | 8 | 0.25 | >64 | >64 | 0.25 |
| PRJEB53700 | 20220649 | *Klebsiella pneumoniae* | 39 | AND11 | VIM-1 | 4 | 2 | 2 | ≤0.06 | 64 | 32 | 0.125 |
| PRJEB53700 | 20220566 | *Enterobacter cloacae* | 63 | AND11 | VIM-1 | >64 | 64 | 64 | 4 | >64 | >64 | 0.5 |
| PRJEB53700 | 20200366 | *Klebsiella pneumoniae* | 70 | AND08 | VIM-1 | >64 | 32 | 16 | 0.5 | >64 | >64 | 0.5 |
| PRJEB53686 | 20200160 | *Enterobacter cloacae* | 78 | AND17 | VIM-1 | >64 | 32 | 16 | 2 | >64 | >64 | 0.5 |
| PRJEB53700 | 20190015 | *Enterobacter cloacae* | 88 | AND05 | VIM-1 | >64 | 64 | 32 | 2 | >64 | >64 | 1 |
| PRJEB53700 | 20210276 | *Enterobacter cloacae* | 90 | AND17 | VIM-1 | 32 | 32 | 8 | 0.25 | >64 | >64 | 1 |
| PRJEB53700 | 20220037 | *Klebsiella aerogenes* | 93 | AND11 | VIM-1 | 16 | 4 | 4 | ≤0.06 | >64 | >64 | 0.25 |
| PRJEB53700 | 20200046 | *Enterobacter cloacae* | 96 | AND01 | VIM-1 | 64 | 32 | 8 | 2 | >64 | >64 | 1 |
| PRJEB53686 | 20190928 | *Citrobacter freundii* | 98 | AND08 | VIM-1 | >64 | 64 | 32 | 4 | >64 | >64 | 1 |
| PRJEB53700 | 20220446 | *Enterobacter cloacae* | 102 | AND17 | VIM-1 | >64 | 64 | 64 | 8 | >64 | >64 | 0.5 |
| PRJEB53700 | 20220133 | *Enterobacter cloacae* | 106 | AND15 | VIM-1 | >64 | 64 | 16 | 2 | >64 | >64 | 1 |
| PRJEB53700 | 20210405 | *Citrobacter freundii* | 111 | AND08 | VIM-1 | >64 | 64 | 16 | 2 | >64 | >64 | 0.5 |
| PRJEB53686 | 20200008 | *Citrobacter freundii* | 116 | AND07 | VIM-1 | 64 | 32 | 16 | 1 | >64 | >64 | 1 |
| PRJEB53700 | 20200387 | *Klebsiella oxytoca* | 135 | AND08 | VIM-1 | >64 | 64 | 32 | 1 | >64 | >64 | 1 |
| PRJEB53700 | 20220277 | *Klebsiella oxytoca* | 145 | AND08 | VIM-1 | >64 | >64 | >64 | 4 | >64 | >64 | 8 |
| PRJEB53700 | 20190836 | *Enterobacter cloacae* | 171 | AND08 | VIM-1 | >64 | >64 | 64 | 4 | >64 | >64 | 0.5 |
| PRJEB53700 | 20220100 | *Enterobacter cloacae* | 175 | AND18 | VIM-1 | >64 | >64 | >64 | 8 | >64 | >64 | 2 |
| PRJEB53700 | 20211053 | *Klebsiella oxytoca* | 190 | AND08 | VIM-1 | >64 | 64 | 64 | 4 | >64 | >64 | 2 |
| PRJNA1133624 | 20210354 | *Klebsiella oxytoca* | 202 | AND08 | VIM-1 | >64 | 64 | 32 | 2 | >64 | >64 | 0.5 |
| PRJEB53700 | 20211121 | *Klebsiella oxytoca* | 226 | AND17 | VIM-1 | 32 | 32 | 8 | ≤0.06 | >64 | >64 | 0.25 |
| PRJEB53700 | 20190024 | *Klebsiella oxytoca* | 237 | AND02 | VIM-1 | 64 | 32 | 16 | 0.5 | >64 | >64 | 1 |
| PRJEB53700 | 20210562 | *Klebsiella pneumoniae* | 307 | AND08 | VIM-1 | >64 | 64 | 32 | 0.5 | >64 | >64 | 0.5 |
| PRJEB53700 | 20210683 | *Enterobacter cloacae* | 311 | AND17 | VIM-1 | 64 | 32 | 16 | 0.5 | >64 | >64 | 0.25 |
| PRJEB53700 | 20190241 | *Enterobacter cloacae* | 344 | AND08 | VIM-1 | >64 | >64 | 64 | 2 | >64 | >64 | 0.25 |
| PRJEB53686 | 20190025 | *Citrobacter freundii* | 432 | AND07 | VIM-1 | >64 | >64 | 32 | 1 | >64 | >64 | 1 |
| PRJEB53700 | 20210026 | *Klebsiella pneumoniae* | 469 | AND17 | VIM-1 | >64 | >64 | >64 | 4 | >64 | >64 | 8 |
| PRJEB53700 | 20200186 | *Enterobacter cloacae* | 523 | AND08 | NDM-1 | >64 | >64 | >64 | 32 | >64 | >64 | 16 |
| PRJEB53686 | 20200043 | *Klebsiella pneumoniae* | 716 | AND01 | NDM-1 | >64 | >64 | >64 | 4 | >64 | >64 | 4 |
| PRJEB53700 | 20210174 | *Enterobacter cloacae* | 742 | AND18 | NDM-1 | >64 | >64 | >64 | 4 | >64 | >64 | 8 |
| CTB: ceftibuten; CTB/A: ceftibuten/avibactam; CTB/L: ceftibuten/ledaborbactam; CTB/X: ceftibuten/xeruborbactam; CAZ: ceftazidime; CAZ/A: ceftazidime/avibactam; MEM: meropenem.  ^a^ EUCAST breakpoints indicated for Enterobacterales | | | | | | | | | | | | |
